# Exploring the Drivers of Food Waste Across EU Member States: A Socio-Economic and Environmental Perspective

**DOI:** 10.3390/foods14244174

**Published:** 2025-12-05

**Authors:** Vardan Aleksanyan, Felix H. Arion, Sargis Gevorgyan, Davit Markosyan, Suren H. Parsyan, Karine Mnacakanyan, Firuta Camelia Oroian, Iulia Cristina Muresan, Iulia Diana Arion, Sabin Chis

**Affiliations:** 1 Faculty of Economics and Management, Yerevan State University, Yerevan 0025, Armenia; v.aleqsanyan@ysu.am; 2 Department of Economic Sciences, University of Agricultural Sciences and Veterinary Medicine of Cluj-Napoca, 400372 Cluj-Napoca, Romania; felixarion@usamvcluj.ro (F.H.A.); camelia.oroian@usamvcluj.ro (F.C.O.); iulia.muresan@usamvcluj.ro (I.C.M.); 3 Faculty of Agribusiness and Economics, Armenian National Agrarian University, Yerevan 0009, Armenia; sargis.gevorgyan17@yahoo.com (S.G.); karine.mnacakanyan.1984@mail.ru (K.M.); 4 Department of Technology Assessment, Leibniz Institute for Agricultural Engineering and Bioeconomy, 14469 Potsdam, Germany; dmarkosyan@atb-potsdam.de; 5 Service-Based Business Management, Armenian State University of Economics, Yerevan 0025, Armenia; surenparsyan2017@gmail.com; 6 Department of Forestry, University of Agricultural Sciences and Veterinary Medicine of Cluj-Napoca, 3–5 Manastur Street, 400372 Cluj-Napoca, Romania; 7 Academy of Romanian Scientists, Ilfov 3, 050044 Bucharest, Romania; 8 Faculty of Food Engineering Tourism and Environment Protection, Aurel Vlaicu University of Arad, 310025 Arad, Romania

**Keywords:** FW, panel data analysis, socio-economic factors, EU-27, sustainability

## Abstract

This study addresses the critical issue of Food Waste (FW) across the 27 European Union (EU) member states by investigating its correlation with key socio-economic and environmental factors. Utilizing panel data regression with a fixed-effects model, this research controls for inherent country-specific characteristics to isolate the influence of variables, such as GDP per capita, educational attainment, environmental taxes, and economic burden on FW levels. The analysis reveals that FW is shaped by a complex interplay of factors, where economic affluence (GDP per capita) and financial stress (housing cost overburden) both exhibit a positive and statistically significant relationship with increased FW. Conversely, higher educational attainment, particularly at the bachelor’s and master’s degree levels, is strongly associated with reduced FW, emphasizing education’s role in promoting sustainable behavior. Environmental policy variables, including environmental taxes and circular material use, are negatively correlated with waste, suggesting effective indirect reduction. Notably, government support for agriculture demonstrates a positive association with FW, potentially indicating incentives for overproduction. These findings highlight the multidimensional nature of FW in the EU, necessitating comprehensive policy responses that integrate educational initiatives, economic levers, and sustainability-oriented reforms to promote resource-efficient consumption across the continent. By clarifying these relationships, this study contributes to the literature by providing one of the few examples of cross-country, EU-wide panel analyses that jointly consider economic, educational, and policy dimensions of FW. The findings offer practical implications for policymakers, emphasizing that FW reduction requires integrated strategies: strengthening environmental taxation and circularity initiatives, aligning agricultural subsidies with sustainability goals, and expanding educational programs that cultivate food-responsible behavior. Together, these insights support the design of more targeted and evidence-based interventions to reduce FW and promote resource-efficient consumption across the EU.

## 1. Introduction

FW is a critical environmental issue that undermines sustainability goals by contributing to greenhouse gas emissions, natural resource depletion, and ecosystem degradation. When food is discarded and left to decompose in landfills, it emits methane (CH_4_), a greenhouse gas that is approximately 25 times more potent than CO_2_ in terms of global warming potential. The environmental burden of FW is not limited to its disposal; it encompasses the entire lifecycle of food—from production to consumption—resulting in excessive water use, energy consumption, and land exploitation. For instance, nearly 30% of global agricultural land is used to produce food that ultimately goes to waste [1,2]

The mismanagement of food also results in substantial water and energy losses. Approximately 250 km^3^ of freshwater is wasted annually in the production of food that is never consumed, intensifying water scarcity in vulnerable regions [2,3]. Furthermore, the energy used in cultivating, processing, packaging, and transporting this food is similarly squandered, leading to inefficiencies across the supply chain and adding to carbon emissions [4,5]. This systemic waste contributes to climate change, with studies revealing that FW management methods such as landfilling and incineration are associated with high emissions of CH_4_ and N_2_O [6,7].

FW is not self-propelling—it is driven by inefficiencies in the supply chain and excessive production. Beyond contributing to climate change, this waste also intensifies biodiversity loss and habitat degradation. Intensive farming practices, often necessary to meet global food demands, contribute to deforestation, soil degradation, and pesticide runoff, which threaten species diversity [2,5]. These ecological effects are compounded when the food produced through such damaging methods is ultimately wasted. FW also exacerbates urban land use pressure, contributing to overcrowding in landfills and increasing the demand for new waste disposal sites [8].

Despite these challenges, FW also presents opportunities for innovation. Circular economy strategies, such as anaerobic digestion, composting, and upcycling, can help recover energy and nutrients from discarded food, reducing environmental damage while creating value from waste [6]. Effective waste management interventions are, therefore, essential not only for environmental protection but also for achieving broader sustainability and food security goals [3,7,8,9,10,11].

The points listed above mainly reflect the consequences that lead to the generation of FW; however, there is a need for a systematic examination of the numerous underlying causes behind FW formation [12]. Despite growing awareness of FW as a major environmental and economic challenge in the EU-27, there is insufficient empirical evidence on how multiple socio-economic and environmental factors influence FW levels across EU-27 countries. Existing studies often examine individual determinants in isolation or rely on cross-sectional data, failing to capture country-specific characteristics and long-term relationships [13,14,15,16].

The objective of this study is to identify and quantify the impact of key socio-economic, educational, environmental, and policy variables on food waste (FW) across the EU-27 member states. Using a random-effects panel data model, the analysis isolates the influence of GDP per capita, educational attainment, environmental taxes, circular material use, housing-cost overburden, and agricultural support on FW levels.

Based on this objective, the study addresses the following research question: *Which socio-economic factors play a significant role in the generation of food waste within the EU-27.*

## 2. Methodology

This section outlines the methodological approach adopted to investigate the relationship between FW and a series of socio-economic and environmental factors across EU member states from 2021 to 2023. The data was collected in 2025 between April and June, making the 2023 results the most recent ones available in Eurostat. The research employs a panel data econometric strategy to account for both temporal and cross-sectional variation and ensure robust, policy-relevant findings.

### 2.1. Data Sources and Variable Description

The study utilizes secondary data collected from Eurostat [17] covering all 27 EU member states for the years 2021–2023. The timespan has been selected considering the emerging essence of reporting FW related data. The dependent variable is FW, measured in kilograms per capita. The independent variables were selected based on theoretical relevance and prior empirical evidence linking them to food consumption patterns, environmental awareness, and socio-economic development. The dataset was prepared following established protocols for panel-data construction to ensure its suitability for econometric analysis. Our objective was to assemble a strongly balanced panel, given the available time period, as balanced structures facilitate more robust estimation and interpretation. To achieve this, missing observations were addressed using a mean-imputation approach, whereby absent values were replaced with the arithmetic mean of the existing observations for the respective country–variable combination. This method, while simple, is commonly applied in empirical panel studies when the proportion of missingness is low and when the underlying data exhibit relatively stable year-to-year variation [18,19]. For consistency and interpretability, all monetary variables were adjusted for inflation where relevant, and variables exhibiting skewed distributions were subjected to natural logarithmic transformation. This transformation helps reduce heteroscedasticity and allows for elasticities to be interpreted directly from the regression coefficients.

The following socio-economic and environmental variables shown in the Table 1, are included in the analysis.

**Figure 1:**
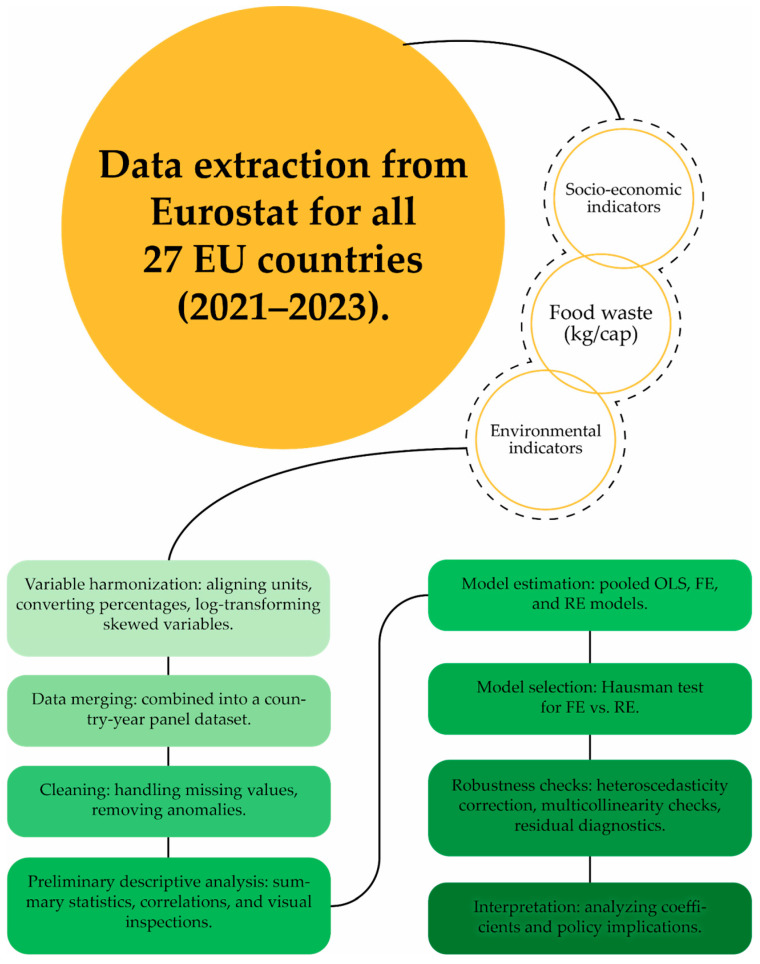
Data collection and analysis process. (Designed by the authors).

The process of data collection and analysis is illustrated more clearly in Figure 1:

### 2.2. Econometric Model Specification

Given the nature of the dataset—multi-country observations across three years—panel data regression techniques were deemed appropriate. Panel data offers several advantages over cross-sectional or time-series data alone, including increased degrees of freedom, reduced multicollinearity, and the ability to control for unobserved heterogeneity.

The basic econometric model can be expressed as:

Υ_it_ = β_0_i + β_1_X_1,it_ + β_2_X_2,it_ + … + β_n_X_n,it_ + ω_i_ + ε_it_

**Element**

**Description**

Υ_it_
Dependent variable for country i at time t. 
β_0i_
Country-specific intercept term capturing unobserved heterogeneity. 
β_1_…β_n_
Coefficients measuring the effect of each independent variable. 
X_1_…X_n_
Independent (explanatory) variables for country i at time t (e.g., GDP per capita, education level). 
ω_i_
Unobserved country-specific effects (fixed or random effects depending on model)
ε_it_
Idiosyncratic error term for country i at time t, assumed to be white noise

Three model types were estimated in the study. First, we applied the pooled OLS model, which assumes homogeneity across countries and time, ignoring individual effects. Fixed effect models, on the other hand, control for all time-invariant characteristics by allowing each country to have its own intercept. Random effects model (RE) assumes that individual effects are random and uncorrelated with the regressors.

### 2.3. Model Selection and Robustness Testing

To determine the most suitable model, we conducted the Hausman Test. This test evaluates whether the unique errors (individual effects) are correlated with the regressors. The null hypothesis favors the Random Effects model, while a rejection indicates that the Fixed Effects model is more consistent. Given the possibility of heteroscedasticity and intra-panel correlation, we employed robust standard errors (also known as Huber–White or sandwich estimators) to ensure that the standard errors of the estimated coefficients are unbiased and efficient. This correction improves the reliability of statistical inference by adjusting for potential violations of the homoskedasticity assumption. Where needed, variables exhibiting high collinearity were considered for exclusion or transformation. To determine the most suitable model specification for our panel data analysis, we performed the Hausman test to compare the fixed effects and random effects models. The test yielded a chi-square statistic of 2.019 with a *p*-value of 0.846. Since the *p*-value is well above the conventional threshold (*p* > 0.05), we fail to reject the null hypothesis that the unique errors are uncorrelated with the regressors. Therefore, the random effects model is preferred, as it provides efficient and consistent estimates in this context.

## 3. Results

### Correlation Matrix and Multicollinearity Assessment

**Figure A1:**
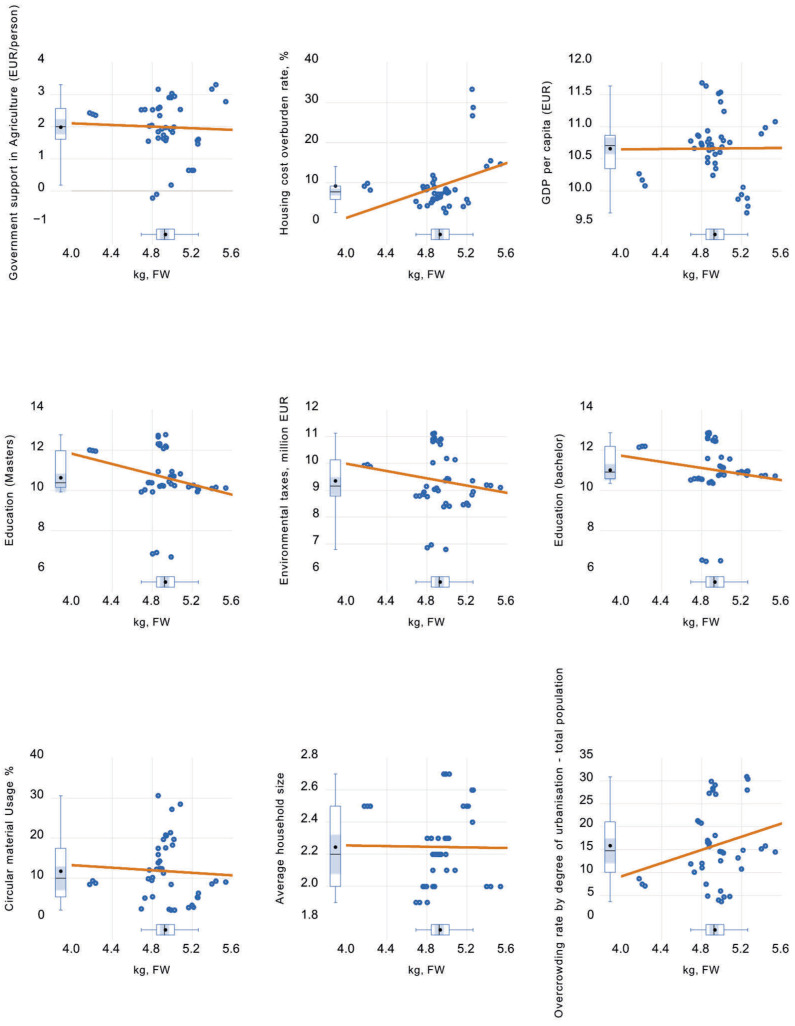
Scatter Plot Map of the Variables (developed by the authors).

Before conducting regression analysis, a Pearson correlation matrix was examined to identify potential multicollinearity among the independent variables. The correlation coefficients are presented in Figure A1 (see Appendix A). Most variables exhibit low to moderate correlations, indicating a relatively low risk of multicollinearity. However, a few variable pairs show strong correlations, which warrant further attention, such as the educational attainment and environmental taxes.

Although these independent variables exhibited high pairwise correlations—notably between *Educational Attainment* (Bachelor’s and Master’s) and *Environmental Tax Revenues*—these variables capture conceptually distinct domains (social capital vs. environmental policy) and were therefore retained in the model. No other variables exceeded the conventional multicollinearity concern threshold (r > 0.8) and, thus, were retained in the analysis. Additionally, government support in agriculture shows moderate correlation with GDP per capita, possibly reflecting the greater fiscal capacity of wealthier nations to support primary sectors. Overcrowding rate is negatively correlated with GDP and positively correlated with housing overburden, suggesting affordability challenges in less affluent regions. Circular material usage shows a moderate positive relationship with population density, which may indicate stronger circular economy practices in more urbanized settings. Overall, the matrix suggests interrelated socio-economic conditions that may indirectly shape FW behaviors across EU member states.

The following Table 2 presents the results of three econometric models estimating the determinants of FW (or the dependent variable of interest) across EU member states: Fixed Effects (FE), Random Effects (RE), and Pooled Ordinary Least Squares (OLS).

The Random Effects model indicates several socio-economic, educational, and policy factors that significantly influence FW across EU member states, and the scatter plot map of the variables can be found in Figure A1 (Appendix A). For improved interpretability of the correlation matrix and the subsequent empirical results, the descriptive statistics are presented below in the Table 3.

*Economic indicators:*

Among economic indicators, Housing Cost Overburden shows a positive and significant effect (5.279, *p* < 0.05), suggesting that households experiencing greater financial strain tend to generate more FW, potentially due to inefficiencies in food management under economic stress. Similarly, GDP per Capita shows a positive and significant association (0.204, *p* < 0.01), indicating that wealthier countries generally produce higher levels of FW, which may reflect increased consumption patterns and lower incentives for careful food management. The unemployment rate, in contrast, is not statistically significant, implying no clear effect on FW within this sample.

*Socio-political and Environmental indicators:*

Educational attainment appears to play a protective role. Higher levels of education are associated with lower FW, with bachelor’s degree attainment showing a significant negative coefficient (−0.253, *p* < 0.05) and master’s degree attainment also negatively associated (−0.134, *p* < 0.05). These findings suggest that better-educated populations may possess greater awareness and skills to optimize food use and reduce waste. Policy-related variables also demonstrate notable effects. Environmental Taxes negatively and significantly impact FW (−0.114, *p* < 0.05), suggesting that higher environmental tax revenues may reflect stronger regulatory frameworks or increased public environmental consciousness. Conversely, Government Support in Agriculture is positively associated with FW (0.922, *p* < 0.01), possibly indicating that subsidies encourage surplus production or less efficient supply chains. Circular Material Usage shows a marginally significant negative effect (−3.995, *p* < 0.1), suggesting that adoption of circular economy practices contributes to FW reduction, supporting sustainability objectives. Finally, demographic factors show mixed results. Average Household Size and Overcrowding Rate are not significant, indicating limited influence on FW in this context. However, Population Density is positively and significantly associated with FW (0.357, *p* < 0.01), suggesting that urbanized, densely populated areas may generate more FW due to consumption patterns and distribution inefficiencies.

## 4. Discussion

Before interpreting the substantive findings, it is important to briefly contextualize the relationship among the different panel estimators used in this study. As presented in Section 3, the coefficients of the random-effects (RE) model align closely with those of the pooled OLS model. This similarity stems from established properties of panel-data estimators: the RE estimator represents a weighted combination of the within (fixed-effects) and between (OLS) estimators [18,19]. When country-specific effects exhibit relatively low variance, or when key explanatory variables display limited within-country variation over time—as is the case for GDP per capita, environmental taxation, and educational attainment—the RE weights converge toward the between estimator, producing coefficients similar to pooled OLS [20]. In contrast, fixed-effects estimation relies solely on within-country variation and becomes less reliable for slowly changing variables, often yielding inflated standard errors and reduced statistical significance. Given these conditions, the RE specification offers the most appropriate balance for interpreting the empirical relationships identified in this study. The detailed overview of the three econometric models can be found in the Table A1.

### 4.1. Economic Indicators (Housing Cost Overburden and FW and GDP per Capita)

The positive and statistically significant relationship between housing cost overburden and FW observed in our analysis aligns with a growing body of literature examining the paradoxical nature of FW in low-income households. Intuitively, one might expect that financial constraints would compel households to minimize FW to maximize resource utilization. However, empirical evidence suggests that poverty can, in fact, stimulate FW through several interrelated behavioral and practical factors.

#### 4.1.1. Purchasing Behavior

Low-income families often engage in purchasing patterns that inadvertently increase FW. For example, to optimize limited financial resources, some households buy food in bulk, motivated by potential cost savings. While this strategy aims to reduce per-unit costs, it can lead to over-purchasing beyond immediate consumption needs, causing spoilage and subsequent disposal [21,22]. Additionally, limited access to proper meal planning or food budgeting tools can result in impulsive buying of items that are ultimately unused, contributing further to food loss.

#### 4.1.2. Food Preparation Practices

FW in economically constrained households can also arise from cooking and consumption habits. Over-preparation of meals is a common issue where more food is cooked than necessary, often due to a desire to avoid frequent cooking or to ensure sufficient portions for family members. Cultural norms and personal preferences regarding leftovers can exacerbate this problem, as some households may avoid consuming leftover food, preferring instead to discard it, thus increasing waste volumes.

#### 4.1.3. Storage Issues

A critical factor contributing to FW among low-income households is inadequate food storage. This can be due to both a lack of proper refrigeration infrastructure and insufficient knowledge about effective food conservation techniques, leading to premature spoilage and wastage. These challenges highlight the intersection of economic deprivation with practical limitations in food management.

#### 4.1.4. Broader Socio-Economic Context

Conversely, the literature often emphasizes that FW is more prevalent in affluent societies, where abundant food production, consumer preferences for variety and freshness, and a culture of disposability dominate [23,24]. This suggests that the drivers of FW are multifaceted and context-dependent, with low-income and high-income households exhibiting different waste generation patterns. Our finding that housing cost overburden—an indicator closely tied to poverty and economic stress—correlates positively with FW underlines the complexity of this issue and the necessity to consider socio-economic heterogeneity in FW reduction policies.

#### 4.1.5. GDP per Capita (EUR)

The random effects model explored the relationship between economic status and FW. The results showed a statistically significant positive correlation between average GDP per person (in euros) and the amount of FW, with a correlation coefficient of 0.204 (** *p* < 0.01). This finding indicates that regions with higher income levels tend to generate more FW, possibly due to increased purchasing power and a greater tendency toward overconsumption or less sensitivity to food loss. To better understand the influence of economic factors on FW, it is important to look at the overall GDP distribution among the countries analyzed. Among the EU-27, Luxembourg, Ireland, and Denmark ranked as the top three countries with the highest average GDP per capita, reflecting strong economic performance and high standards of living. In contrast, the bottom three countries, Bulgaria, Romania, and Croatia, reported the lowest GDP per capita figures, indicating more limited economic resources. These disparities in economic strength may partially explain the variation in FW behavior across countries. Higher GDP levels in nations like Luxembourg and Ireland may enable consumers to purchase more food than necessary, potentially causing greater waste. On the other hand, in lower-income countries, such as Bulgaria and Romania, financial constraints might facilitate more cautious purchasing habits and better food utilization. These socio-economic contrasts provide important context for interpreting the statistically significant positive correlation (r = 0.204, ** *p* < 0.01) found between GDP and FW in the random effects model. As reported in the United Nations Environment Program’s FW Index 2021, households in high-income countries with elevated GDP levels generate, on average, 79 kg of FW per person each year, along with 26 kg per capita from food services and 13 kg per capita from the retail sector [25]. An analysis of ten studies conducted across various regions and income levels reveals that household FW often tends to rise with increases in per capita GDP. For example, research found that a 10% growth in per capita income corresponds to a 7% increase in household FW, a result statistically significant at the 95% confidence level [26]. Another research identified a log-linear relationship, estimating a global affluence elasticity of 1.09 [27]. Similarly, another study observed a shift in China from a slight negative correlation during the 1990s to a strongly positive link in the 2000s [28]. However, some studies report different patterns where authors did not find a consistent association [29]. In high-income countries, annual per capita waste ranges between 34.33 kg and 109.3 kg [30,31]. In contrast, middle-income countries report considerably lower figures, such as 14.9 kg in China and 7.3 kg in South Africa [28]. These observations suggest that while economic prosperity is often linked with higher levels of household FW, the relationship is not completely linear across all countries and income categories.

### 4.2. Socio-Political and Environmental Indicators (Education Level (Bachelor’s and Master’s Degree Environmental Taxes, Overcrowding Rate and Population Density)

Our analysis using a random effects model showed a meaningful connection between education and FW. A statistically significant negative correlation was found between the amount of FWd (in kilograms) and the proportion of individuals holding a master’s degree (r = −0.134, *p* < 0.05). Similarly, a negative correlation was observed between FW and the proportion of individuals with a bachelor’s degree (r = −0.253, *p* < 0.05), further supporting the role of education in reducing FW. In simpler terms, areas with a higher percentage of educated individuals tend to waste less food. This trend is also reflected in the scatter plot with the regression line, which clearly illustrates that higher educational attainment often goes hand in hand with more responsible food behavior. This could be due to better planning, increased awareness of FW issues, or a stronger sense of environmental responsibility among those with higher education. However, these findings stand in contrast to those of Bilska et al., whose study on Polish consumers reported that individuals with university-level education were more likely to make unplanned purchases and waste food. According to the authors, this points to a potential link between higher education and increased impulsivity in food buying behavior, which may contribute to greater FW in certain contexts [32]. Research underlines that, not only in the European context but also across Asian countries, such as in Indonesia, environmental literacy and food-waste-oriented education play a vital role in addressing the issue in the long term. However, it is important to recognize that education alone is not the determining factor influencing the FW phenomenon [33]. Effectively tackling FW requires a systemic approach, as the socio-economic drivers underlying FW are deeply interconnected [33,34].

***Environmental taxes:***

The panel analysis reveals that environmental taxes are negatively associated with FW levels, particularly under the random and pooled effects models, where the coefficients are statistically significant (random effect: −0.114 **, pooled effect: −0.114 ***). However, the fixed effect coefficient (−0.0373) is smaller and not statistically significant, suggesting that country-specific unobserved characteristics may moderate the relationship between environmental taxes and FW. Different studies also confirm that countries with higher environmental taxes do not necessarily experience a proportionate reduction in FW. This can be attributed to the broader design of environmental tax regimes, which are primarily aimed at reducing emissions, promoting cleaner production, and improving overall environmental performance, rather than directly targeting FW. Indeed, prior research supports this distinction. For example, environmentally related taxes contribute to the reduction of CO_2_ emissions, PM10 levels, and fossil fuel consumption, demonstrating a clear positive impact on general environmental performance [35]. However, the direct influence of these taxes on FW reduction remains limited. The nature and structure of the taxes play a critical role. General environmental taxes may exert only an indirect influence on FW by encouraging sustainable behaviors or penalizing environmentally harmful activities. Without specific provisions or incentives targeting FW, their impact remains diffuse and uncertain. Recent literature has started to explore how targeted tax policies can influence FW reduction. For instance, tax incentives for food donations to social economy entities can act as a facilitator in mitigating FW [36]. Similarly, another research argues that modifying VAT reductions, such as removing them from high-impact food items like meat, could alter consumer behavior and reduce environmentally intensive food consumption, thus indirectly curbing FW [37].

Nonetheless, policy implementation remains a challenge. The study discusses the theoretical potential of optimal food-waste disposal taxes, yet highlights the scarcity of practical, operational frameworks for such taxes [38]. Further research suggests that effective reductions in FW are unlikely to result from fiscal measures alone. Instead, a multifaceted approach—combining targeted taxation, public awareness campaigns, food redistribution programs, and waste prevention initiatives—is essential [39].

***Overcrowding rate and Population density:***

Recent academic research increasingly emphasizes the intricate relationship between urbanization and the rise in FW, particularly in low- and middle-income countries. High levels of urban development in these regions often lead to a surge in both food production and consumption, thereby contributing to a larger share of FW within the overall waste stream. This trend is especially visible in urban households, retail sectors, and among informal food vendors. As urban areas expand and population density intensifies, managing FW becomes progressively complex, requiring the implementation of more advanced and adaptive waste management systems. According to various studies, urban centers are expected to witness a substantial increase—up to 35%—in urban FW between 2007 and 2025, driven by economic growth and demographic pressures [16]. Moreover, our own findings reveal a significant positive correlation between population density (per square kilometer) and the overcrowding rate, reinforcing the link between spatial concentration and waste generation. As more than half of the world’s population now resides in urban areas, these challenges demand urgent and effective policy interventions [40]. Yet, urbanization also opens pathways for sustainable innovations such as composting, anaerobic digestion, and circular solutions, which, if strategically integrated, could counterbalance the negative externalities of increasing FW and support broader sustainability goals, including those outlined in the UN Sustainable Development Agenda [41].

## 5. Conclusions

This study investigated the socio-economic and environmental determinants of FW across the EU-27 using panel data regression analysis. The findings reveal that housing cost overburden, GDP per capita, educational attainment, environmental taxes, and circular material usage significantly influence FW generation. In particular, the positive association between housing cost overburden and FW suggests that financial stress may paradoxically contribute to inefficient food practices, such as bulk purchasing, inadequate storage, and poor meal planning. Higher GDP per capita correlates with increased FW, reflecting patterns of overconsumption and disposability in affluent societies, whereas higher educational attainment significantly reduces FW, highlighting the potential of education to foster sustainable consumption behaviors. Environmental taxes and circular economy practices indirectly support FW reduction, while agricultural subsidies may inadvertently encourage overproduction and inefficiencies.

These findings carry important implications for policy and practice. Effective FW reduction strategies require integrated, multidimensional approaches that combine targeted educational campaigns, fiscal measures promoting sustainable behavior, and reforms in agricultural subsidy frameworks. At the consumer and market level, insights from this study support the development of initiatives such as food-sharing platforms, donation incentives, and digital tools for meal planning and food tracking, which can reinforce responsible consumption patterns.

Despite these contributions, this study has several limitations. Only selected socio-economic, environmental, and policy variables were considered, leaving out behavioral, cultural, and technological factors that may also shape FW. Moreover, mean imputation was used for missing data, which, while standard, may not fully capture temporal variability. Future research should incorporate additional behavioral and cultural indicators, explore country-specific intervention effects, and examine the effectiveness of policy and market instruments such as food redistribution programs or digital consumer tools across different socio-economic contexts.

Overall, this study underscores the fact that reducing FW in the EU is not solely an environmental challenge but also a socio-economic and behavioral one. Targeted, evidence-based interventions that consider household behavior, market dynamics, and policy instruments are essential to promote sustainable food practices and more efficient resource use across Europe.

## Figures and Tables

**Table 1:** List of the Variables included in the study.

Variable	Description	Justification for Selection
**FW**	Amount of FWd per capita	Main dependent variable of the study, representing the core issue under investigation.
**Housing Overburden Rate**	% of population spending 40%+ of disposable income on housing	Indicates financial pressure, which may limit capacity for efficient food consumption/storage.
**GDP per Capita (EUR)**	National economic output per person	Reflects affluence and living standards; may influence both overconsumption and food management.
**Education (Bachelor)**	% of population aged 25–64 with a bachelor’s degree	Higher education often leads to greater environmental and nutritional awareness.
**Education (Master)**	% of population aged 25–64 with a master’s degree	Further educational attainment may enhance sustainable consumption and planning habits.
**Unemployment Rate**	% of labor force that is unemployed	Economic insecurity may impact food purchasing and waste behavior unpredictably.
**Environmental Taxes**	Revenue from environmental taxes (in million EUR)	Indicates the strength of environmental policy; may influence societal behavior around waste.
**Government Support in Agriculture**	State financial support to agriculture sector (in million EUR)	Affects food production systems and potential surplus, indirectly influencing FW.
**Circular Material Usage**	% of material input from recycled sources	Reflects circular economy practices; higher rates may correlate with better waste management.
**Average Household Size**	Number of people per household	Larger households may exhibit more efficient food usage per capita.
**Overcrowding Rate**	% living in overcrowded housing, by urbanization level	Linked to limited storage and food planning capacity, potentially increasing FW.
**Population Density**	People per square kilometer	High density may affect waste infrastructure and individual waste behavior.

**Table 2:** Correlation Matrix (developed by the authors).

	−1	−2	−3	−4	−5	−6	−7	−8	−9	−10	−11
**(1) Housing Overburden Rate**	1.0										
**(2) GDP per Capita (EUR)**	0.0	1.0									
**(3) Education (Bachelor’s)**	0.1	0.0	1.0								
**(4) Education (Master’s)**	0.1	0.0	0.9	1.0							
**(5) Unemployment Rate**	0.4	−0.1	0.0	0.1	1.0						
**(6) Environmental Taxes (mil EUR)**	0.2	0.1	0.9	0.9	0.0	1.0					
**(7) Government Support in Agriculture**	0.1	0.4	0.1	0.1	0.0	0.2	1.0				
**(8) Circular Material Usage (%)**	0.0	0.2	0.3	0.3	−0.1	0.4	0.2	1.0			
**(9) Average Household Size**	−0.1	−0.3	−0.1	−0.1	0.0	−0.3	−0.4	−0.3	1.0		
**(10) Overcrowding Rate**	0.2	−0.5	0.0	0.0	0.0	−0.1	−0.4	−0.3	0.2	1.0	
**(11) Population Density (km^2^)**	−0.1	0.1	0.0	−0.1	−0.3	0.0	−0.1	0.5	0.0	−0.4	1.0

**Table 3:** Descriptive Statistics of Variables.

Variable	Obs	Mean	Std. Dev.	Min	Max
FW, kg	81	137.099	47.168	65	294
Housing cost overburden, %	81	7.344	5.424	1.9	33.3
GDP per capita, EUR	81	34,486.914	23,182.057	9450	118,310
Education bachelor’s degree holders	81	80,624.333	102,616.65	643	388,732
Education Master’s degree holders	81	55,968.123	79,708.015	801	351,459
Education Ph.D. degree holders	81	3501.235	5644.399	66	28,153
Unemployment rate, %	81	4.21	1.778	1.5	10
Environmental taxes, EUR	81	11,717.242	16,972.673	276.58	68,238.328
Government support in Agric., EUR	81	7.322	5.892	0.1	27.3
Circular material usage, %	81	10.223	6.808	1.3	30.6
Average household size	81	2.342	.28	1.9	3.1
Overcrowding rate, %	81	21.141	13.186	1.9	47.8
Population density km^2^	81	185.637	312.217	18.2	1692.7

## Data Availability

The original contributions presented in this study are included in the article. Further inquiries can be directed to the corresponding authors.

## Appendix B

**Table A1:** The results of three Econometric models estimation, (FE), (RE), and (OLS). (Developed by the authors).

	(1)	(2)	(3)
	Fixed Effect	Random Effect	Pooled OLS
Housing Cost Overburden	1.133	5.279 **	5.279 **
	(5.351)	(2.203)	(2.629)
GDP Per Capita (EUR)	0.0480	0.204 ***	0.204 ***
	(0.0621)	(0.0385)	(0.0616)
Education Bachelor	−13.74	−0.253 **	−0.253 *
	(23.69)	(0.122)	(0.145)
Education Masters	−0.0541	−0.134 **	0.134 *
	(0.0800)	(0.0597)	(0.0761)
Unemployment Rate	1.937	7.522	7.522
	(1.622)	(5.980)	(7.792)
Environmental Taxes (million EUR)	−0.0373	−0.114 **	−0.114 ***
	(0.0831)	(0.0445)	(0.0389)
Government support in Agriculture	0.444	0.922 ***	0.922 ***
	(0.316)	(0.243)	(0.170)
Circular Material Usage	1.135	−3.995 *	−3.995 *
	(1.48)	(2.11)	(2.072)
Average Household Size	−1.070	5.078	5.078
	(1.208)	(4.031)	(4.87)
Overcrowding Rate	1.543	−9.938	−9.938
	(3.054)	(1.364)	(1.175)
Population Density (People per km^2^)	−0.0332	0.357 ***	0.357 ***
	(0.116)	(0.124)	(0.0862)
_cons	−1.28845 × 10^9^	−1.07914 × 10^9^	−1.07914 × 10^9^
	(5.79563 × 10^9^)	(875,562,305.6)	(1.20108 × 10^9^)

Standard errors in parentheses. * *p* < 0.10, ** *p* < 0.05, *** *p* < 0.01.
